# Optimizing the prediction of discard survival of bottom-trawled plaice based on vitality indicators

**DOI:** 10.1093/conphys/coae070

**Published:** 2024-10-09

**Authors:** Sven Sebastian Uhlmann, Esther Savina, Junita Karlsen, Bart Ampe

**Affiliations:** Fisheries and Aquatic Production, Animal Sciences Unit Flanders Research Institute for Agriculture, Fisheries and Food (ILVO), Jacobsenstraat 1, 8400 Ostend, Belgium; Marine Institute, Rinville West, Rinville, Co, Galway, H91 R673, Ireland; Marine Biology - Ecology, Evolution & Genetics, Vrije Universiteit Brussel (VUB), Pleinlaan 2, 1050 Brussels, Belgium; Technical University of Denmark National Institute of Aquatic Resources, North Sea Science Park, Willemoesvej 2, 9850 Hirtshals, Denmark; Technical University of Denmark National Institute of Aquatic Resources, North Sea Science Park, Willemoesvej 2, 9850 Hirtshals, Denmark; Technical University of Denmark National Institute of Aquatic Resources, North Sea Science Park, Willemoesvej 2, 9850 Hirtshals, Denmark; Fisheries and Aquatic Production, Animal Sciences Unit Flanders Research Institute for Agriculture, Fisheries and Food (ILVO), Jacobsenstraat 1, 8400 Ostend, Belgium

**Keywords:** Catch welfare, indicator optimization, landing obligation, reflex action mortality predictor (ramp)

## Abstract

Predicting the discard survival of aquatic animals after fisheries capture using vitality indicators (i.e. individual scores or indices of physical condition) is a resource-efficient approach compared to estimating discard survival from captive observation. But such indicators do not always lead to accurate and robust predictions. Individual scores of reflex impairments and injuries are typically given the same weight when being aggregated into an index, while some reflexes or injuries may contribute to mortality more than others. This study established an analytical methodology and created an index based on differential contributions of individual reflexes and injuries to optimize the prediction of discard survival of bottom-trawled European plaice (*Pleuronectes platessa*). The optimization procedures were applied to a dataset from vitality assessment of 1122 undersized plaice caught during 16 commercial fishing trips and 58 gear deployments in Belgium and Denmark. As welfare indicators, we considered and evaluated against post-capture survival of plaice: original vs. optimized reflex impairment and injury (R&I) index, number of absent reflexes, number of present injuries, number of absent reflexes and present injuries, categorical vitality score and individual reflex and injury scores. These were used in eight candidate generalized linear models (one without any vitality indicator) as explanatory variables to predict survival, with or without biological, environmental, technical and operational covariates, either at the individual fish or trip level. Bruising to the head and body were the most relevant predictors. The optimized R&I index did not perform better than any other vitality indicator, and all the indicators performed poorly in predicting survival probability both at the fish and trip levels without information on air exposure and seawater temperature. This means that they cannot be considered to be independent measures. The categorical vitality score provided a viable alternative to the more labour-intensive, scoring method of reflex responsiveness. Use of reflexes as proxies may not be accurate when they are not independent of environmental, biological or technical variables.

## Introduction

In response to fisheries management policies requiring a quantifiable measure for post-release (discard) fate of fish, survival estimates at the level of individual fish, fleets or fisheries are generated from captive observations (Raby *et al*., 2012; Brownscombe *et al*., 2017; Rihan *et al*., 2018) or tagging studies (Morfin *et al*., 2019a). Scaling up from individual to population level requires the use of reliable proxies (Horodysky *et al*., 2015). But these can show variation given the complexities of capture and handling processes and the inherent sensitivity of organisms. Physiological indicators of primary and secondary stress responses typically fail to predict survival (in captivity) given the complexities of physiological responses and different recovery capabilities of animals and individuals (Wood *et al*., 1983; Brownscombe *et al*., 2017). Alternatively, tertiary stress responses such as vitality indicators can be more cost-efficient to collect in remote and adverse field environments but can be equally unreliable as predictors of post-release survival (Kraak *et al*., 2019; Lennox *et al*., 2024).

Vitality is a measure of an organism’s health (Breen and Catchpole, 2021) and measuring whole-animal responsiveness to a stimulus (or reflex robustness) can be used as an indication of neural integrity (Davis and Ottmar, 2006; Davis, 2010; Sopinka *et al*., 2016). Reflex responses are central pattern generators that are involuntary motor responses to pre-defined external stimuli and which do not require any output from the brain (Grillner, 1996). The concept assumes that an animal’s ability to respond to stimuli (sometimes considered together with the extent of external injury), integrates the effects of multiple stressors. These can be biological (e.g. sex, size, age), environmental (e.g. temperature), technical (e.g. gear design) and operational (e.g. fishing depth, fishing duration) stressors to which an animal is exposed throughout the catch-and-discarding process (Davis and Ottmar, 2006; Davis, 2010). While fishing capture-related physical impact can cause injuries, it can also trigger a primary or secondary stress response. The pathway of nerve impulses from the receptors to the muscles through the brainstem and spinal cord (Roberts, 1986; Hildebrandt *et al*., 2015) might be affected by stress via an altered metabolism from anaerobic exercise and hypoxia which in turn may lead to impaired reflexes (Davis, 2002; Forrestal *et al*., 2017). It is thus assumed that reflex responses mirror internal physiological stress responses resulting from injuries (e.g. Olsen *et al*., 2012), exhaustion and fatigue from anaerobic exercise during herding and crowding (Holder *et al*., 2022), hypoxia or changes in metabolic rates with fluctuations in temperature (Davis, 2002; Davis, 2010; Forrestal *et al*., 2017).

Vitality assessments are popular in the European Union (EU) to provide an indicator for post-capture survival of flatfish (e.g. Uhlmann *et al*., 2016; Morfin *et al*., 2017, Morfin *et al*., 2019; Kraak *et al*., 2019) attributed to fishing stressors in supporting exemptions to the landing obligation article 15 (Uhlmann *et al*., 2019). Due to the sheer quantity of species-fishery combinations, it is not feasible to observe discard survival for all the existing métiers within the limited resources available, and methods for reliable survival predictions for other fleet segments and/or fishing areas where related fleets are fishing are needed (Scientific, Technical and Economic Committee for Fisheries (STECF), 2018; Scientific, Technical and Economic Committee for Fisheries (STECF), 2021). The method has become even more relevant since predictions of survival probability from on-board vitality assessments without observations of delayed mortality after discarding have become sufficient as management input to obtain a high survival exemption from the EU Landing Obligation (Rihan *et al*., 2018; STECF, 2021). For example, reflex impairments have been used to predict post-capture survival of European plaice (*Pleuronectes platessa*) discarded from a French otter-trawl fishery. But as observations of post-capture survival were lacking, this was instead inferred based on a modelled relationship between vitality and post-capture survival of discarded plaice from a similar English fishery (Morfin *et al*., 2017). To make predictions within and across fleet segments or across different fishing areas, the assumption that the severity of the stressors is precipitated in the chosen vitality indicator must be fulfilled. Furthermore, the underlying relationships between impairment of reflexes or occurrence of injuries and survival must be immutable and consistent, which may not necessarily be the case (Morfin *et al*., 2017; Morfin *et al*., 2019; Kraak *et al*., 2019). Variations in the conditions (e.g. temperature, air exposure) under which a fish is tested for vitality, including if they are allowed to recover (e.g. by being held in water-filled containers while waiting for assessment, similarly to having running seawater in the pounder) may influence both reflex impairment and their survival and so the ability of the indicator to predict survivability.

A vitality indicator can be expressed as a simple proportion of impaired reflexes, present injuries or as an index generated from impaired reflexes and present injuries scores (Meagher, 2009; Davis, 2010). The latter implies, however, that both reflex impairment and injuries contribute with equal weights to post-capture survival and has thus been criticized for disregarding any differential contributions of individual reflexes to the observed mortality (Breen and Catchpole, 2021). To test whether some reflexes and injuries may be more relevant than others for the survival of the fish, the performance of different optimization functions can be evaluated to optimize the weightings of individual reflex and injury attributes. Even if the survival of a fish can be predicted with reasonable certainty based on the observed reflex impairment and/or injuries or categorical vitality scores, predicting mean survival at the trip level can become challenging. Similar mean scores may be obtained for different trips despite different scores for different vitality attributes (e.g. if one trip gives high score for reflexes and low for injuries and vice versa for another trip so the two effects cancel each other out; Uhlmann *et al*., 2021). This situation is more likely if all reflexes and injuries are given equal weight. A reliable vitality indicator should therefore be optimized not only at the fish level but also at the trip level.

In this study, we aimed at (i) optimizing a reflex and injury (RI_optimized_) index to test (ii) whether it improved discard survival predictions of bottom-trawled plaice compared with six other indicators, (iii) how well it predicted the discard survival estimated from captive observation and (iv) if the predictions improved when adding covariates (i.e. gear type, gear deployment duration, catch weights, fishing depth, seawater temperature and air exposure). The six other vitality indicators were RI index, number of impaired reflexes, number of present injuries, number of impaired reflexes and present injuries, categorical vitality score, individual reflex and injury scores. The tests were done for individual fish and at the trip level with the intention to develop a generic methodology that can be used in comparable contexts of using vitality indicators to predict discard survival.

## Materials and Methods

### Fishing operations

Vitality and discard survival data of undersized, trawl-caught plaice were collected following a harmonized protocol (Uhlmann *et al*., 2016, 2021; Breen and Catchpole, 2021) from four Belgian beam trawlers and one Danish otter trawler, respectively. The vessels were fishing under conditions representative of their respective demersal bottom trawling fleets with regards to engine capacity and target species. The double-rigged Belgian beam trawlers (vessel power > 221 kW) targeted common sole (*Solea solea*) in the eastern North Sea, eastern and western English Channel and northwestern Atlantic Ocean. These vessels were rigged with two ~ 12-m beam trawls with chain mats (each weighing between ~ 4750 and ~5500 kg), a body made of either nominal 120 mm or 150 mm diamond mesh, and nominal 80 mm diamond-mesh codends. The Danish commercial otter trawler (vessel power 217 kW) targeted plaice in the western Baltic Sea (ICES area 24) and was fished with either two nominal 120 mm T90 mesh codends, or two nominal 105 mm diamond mesh with nominal 120 mm BACOMA panels in a twin-rig. Biological (fish length), environmental (surface seawater temperature), technical (gear design) and operational variables (fishing depth, gear deployment duration, total catch weight, air exposure) were collected on board all vessels at the trip, deployment or fish level (Table 1). The sea surface temperature and water temperature in all holding containers was monitored with a handheld YSI Pro 2030 multi-parameter (Belgium) or Handy Polaris 2 (Oxyguard, Denmark) sensors to document that plaice were held under temperature conditions mimicking their natural environment. All loggers were calibrated and maintained following recommended manufacturer’s procedures. Summaries of key technical, environmental and biological variables collected during each monitored trip were published in Uhlmann *et al*. (2016, 2021, 2023) for Belgian trips and in Savina *et al*. (2019, 2024) for Danish trips.

**Table 1:** Description of the response (mortality at asymptote) and explanatory variables (covariates and vitality indicators) that were collected for trawl-caught European plaice (*Pleuronectes platessa*) either at the trip, gear deployment (haul) or fish level

Level	Variable	Description	Type
Trip	Trip	Unique trip ID from 1 to 14 in chronological order	Categorical
Trip	Gear type	Beam-trawl (BT2, Belgium) or otter-trawl (OTB, Denmark) gears	Categorical
Deployment	Seawater temperature (°C)	Surface seawater temperatures were measured with a YSI multi-parameter (Belgium) or Handy Polaris 2 (Denmark) probe	Continuous
Deployment	Fishing depth	Average fishing depth (in meters)	Continuous
Deployment	Gear deployment duration	Time period between the start time of the gear deployment, i.e. when the whole trawl was in the water, and the time when the gear arrived on deck (in minutes)	Continuous
Deployment	Total catch weight	Estimate of the total catch weight inside the hopper(s) (in kg)	Continuous
Fish	Air exposure	Air exposure of each tagged fish was expressed as the minutes it spent on deck before being placed inside a water-filled container plus one third of the handling time during reflex testing (because 1/3 of the reflex tests were done in air out of the water-filled container)	Continuous
Fish	Fish length	Total fish length (cm)	Continuous
Fish	Mortality at asymptote	Status (status scored as 0 = alive; or 1 = dead) of a fish once in captivity and at the end of monitoring (at asymptote)	Binary
Fish	Hours alive	The time spent of a fish surviving between being collected from the catch and the time when it was assessed as dead	Continuous
Fish	Body flex (behaviour)^1^	Fish is held outside the water on the palms of two hands (touching each other) with its ventral side facing up and its head and tail unsupported. Response: The fish is actively trying to move head and/or tail towards each other or is wriggling out of the hand	Binary
Fish	Righting (behaviour) ^1^	Fish is held underwater at the surface on the palms of two hands (touching each other) with its ventral side facing up and then slowly released. Response: The fish is actively righting itself under water	Binary
Fish	Head complex (reflex) ^1^	Observing the opening of the operculum and/or mouth for breathing for 5–10 s in air^1^ (scored as ‘operculum’ during the first trip)	Binary
Fish	Evasion (behaviour) ^1^	Fish is held underwater in an upright position close to water surface and then released. Response: The fish swims actively away towards the bottom of the container	Binary
Fish	Stabilize (behaviour) ^1^	This reflex is scored immediately after evasion. No extra handling is required. Response: The free-swimming fish tries to find a good position on the bottom by fixing itself and/or rhythmic and swift movement of the fins and/or body as if it would bury itself into sediment; Response: No visible displacement, resting motionless in one place within 3 s after reaching the bottom following evasion.	Binary
Fish	Tail grab (reflex) ^1^	Fish is gently grabbed by the caudal peduncle between thumb and index finger. Response: Actively struggles free and swims away.	Binary

(Continued)

**Table 1:** Continued

Level	Variable	Description	Type
Fish	Head bruising^2^	Haemorrhage on the head region of a fish. The surface area (%) of the head region covered by haemorrhages was scored on a 4-point categorical scale: absent = 0; < 10% = 1; ≥10–<50% = 2; ≥50% = 3. For the calculation of the R&I index and other vitality indicators, the four-point scale was converted to a binary scale with injury absent (level 0) and injury present (levels 1, 2 and 3)	Binary
Fish	Body bruising^2^	The surface area (%) of the body region covered by haemorrhages was scored on a 4-point categorical scale: absent = 0; < 10% = 1; ≥10–<50% = 2; and ≥ 50% = 3. For the calculation of the R&I index and other vitality indicators, the four-point scale was converted to a binary scale with injury absent (level 0) and injury present (levels 1, 2 and 3)	Binary
Fish	Head point bleeding^2^	Multifocal cutaneous petechiae to the head region of a fish (absent = 0; 1: < 10% surface area coverage; 2: 10–50%; and 3: > 50%). For the calculation of the R&I index and other vitality indicators, the four-point scale was converted to a binary scale with injury absent (level 0) and injury present (levels 1, 2 and 3)	Binary
Fish	Body point bleeding^2^	Multifocal cutaneous petechiae to the body region of a fish (absent = 0; 1: < 10% surface area coverage; 2: 10–50%; 3: > 50%). For the calculation of the RI index and other vitality indicators, the four-point scale was converted to a binary scale with injury absent (level 0) and injury present (levels 1, 2 and 3)	Binary
Fish	Reflex impairment and injury (RI) index	Mean score of impaired reflexes and present injury (sum of impaired reflex and present injury scores divided by the total number of tested reflexes and injury types). This resulted in values ranging between 0 and 1, whereby 0 would represent an unimpaired, i.e. reflex responsive and uninjured fish and 1, represented an impaired and injured fish	Continuous
Fish	Number of impaired reflexes	Sum of impaired reflexes	Continuous
Fish	Number of present injuries	Sum of present injuries	Continuous
Fish	Number of impaired reflexes and present injuries	Sum of impaired reflexes and present injuries	Continuous
^1^Fish	Categorical vitality score	A = Fish in excellent condition, vigorous body movement and no or minor external injuries only B = Fish in good condition, weak body movement, minor external injuries C = Fish in poor condition, no body movement but fish can move operculum and minor/major external injuries D = Moribund or dead, no body or opercular movements	Categorical

### Vitality assessment

To assess vitality, protocol-instructed researchers who all had received the same training, randomly sampled undersized plaice, i.e. below the minimum conservation reference size of 27 cm total length (TL), throughout the catch sorting process at the point of discarding. Plaice were placed into grey, water-filled, 30–90-liters PVC holding containers awaiting assessment. Seawater was refreshed at least every 10 min. During the assessment, grey, water-filled containers of 40 × 60 × 20 cm were used. Each fish was scored for the presence/absence of responses to pre-defined external stimuli, visible bleeding injury and vigour of movements (activity) (for details, see Uhlmann *et al*., 2016, 2021). Six candidate reflexes were *a priori* identified based on earlier experimental work (see Uhlmann *et al*., 2016, 2021; Table 1) and published information (Davis, 2007, 2010; Depestele *et al*., 2014). Reflexes were tested in the following order: ‘body flex’; ‘righting’; ‘head complex’; ‘evasion’; ‘stabilize’ and ‘tail grab’. A visible (weak; see Meeremans *et al.*, 2017 for descriptions of reflex response intensities of a present reflex) response was scored as impairment being absent (score = 0) or present (score = 1) within 5 s of observation. Each plaice was also scored for the severity of four types of injuries: bruising and point bleeding to the head or body (Table 1). Injuries were scored on a four-point categorical scale (absent = 0; < 10% = 1; ≥10–50% = 2; ≥50% = 3), further dichotomized as a binary scale for the purpose of calculating the RI index (injury absent = 0; injury present = 1). For the categorical vitality score, each plaice was assigned one of the four alternative categories ranging from A (excellent condition) to D (dead or moribund) (Table 1). The whole reflexes, injuries and vitality score assessment took < 1 min per fish. All evaluated fish that were unresponsive to any of the reflex tests were recorded as dead. All sampled fish were length-measured to the nearest 1 cm of TL. In Belgium, plaice were T-bar (29 × 8 mm) anchor tagged with Bano’k© guns in the dorsal musculature according to McKenzie *et al.* (2012). In Denmark, plaice were tagged using passive integrated transponder tag (“HPT 12”, Biomark, USA) on the pigmented side in the dorsal muscles to identify individual fish (Savina *et al*., 2019).

### Survival assessment

Alive vitality-assessed fish (3–19 and 12–44 individuals per deployment in Belgium and Denmark, respectively) were then placed into water-filled 24–30 L monitoring containers of a custom-built unit with a maximum of five individual(s) per container. The fish were inspected every 12 hours on board the vessel for between 1 and 8 days and every 6 or 24 hours after transfer to a laboratory holding facility (within 1 hour of road transport). Water temperature at the holding facility was matched as closely as possible to the environmental conditions from which plaice were trawled. However, temperature readings at fishing depth were not available from all operations. Ashore, all monitoring containers were supplied with sand as bottom substrate and fish were fed daily *ad libitum* with a mix of polychaete worms (*Nereis* spp.), blue mussel (*Mytilus edulis*) and uncooked, defrosted brown shrimp (*Crangon crangon*) in Belgium and Atlantic northern shrimp (*Pandalus borealis*), whiting (*Merlangius merlangus*) and Atlantic mackerel (*Scomber scombrus*) in Denmark. The number of hours each fish survived since collection were calculated. The monitoring periods differed between 6 and 34, and 13 and 33 days in Belgium and Denmark, respectively.

### Statistical analysis

In this study, a combined dataset of trawled-and-discarded plaice from Denmark and from Belgium was used to identify which vitality indicator gave the best prediction of discard survival of bottom-trawled plaice at the individual fish and fleet level. Each candidate generalized linear model (GLM) was built with mortality at asymptote (at the fish level with 0 for alive and 1 for dead) as the response variable. Mortality of asymptote was determined when the mortality curve flattened before the end of the monitoring period. The fish affected by capture and discarding are expected to die within the first few hours or days, whereas the fraction of individuals unaffected by capture and discarding are expected to die according to their normal life schedule, i.e. on the scale of years. A near zero probability of death by natural causes is assumed during the holding period, i.e. on the scale of days. The overall observed survival is thus a combination of a rapid decline followed by a constant survival rate (Benoît *et al*., 2012).

In addition, all models were tested with the coherent biological, environmental, technical and operational explanatory variables (covariates), i.e. fish length, surface seawater temperature, gear type, fishing depth, gear deployment duration, total catch weight, air exposure and two plausible interactions, one between gear type and surface seawater temperature and the other between air exposure and surface seawater temperature. Random effects were not considered for these predictive models because it was not relevant to predict to a random variable such as deployment, day or trip, and the aim of this study was to find the best predictive model, not to identify significant contributing effects of explanatory variables. We used logistic regression models (GLMs) as follows:


\begin{equation*} {Y}_{ij}\sim Bin\left(1.{p}_{ij}\right)\ \end{equation*}

$$ \eta \left({p}_{ijk}\right)=\alpha +{\beta}_1{X}_{1. ijk}+{\beta}_2{X}_{2. ijk}+\dots +{\beta}_n{X}_{nijk}+{e}_{ijk} $$

where $\eta$ represents the logit-link function; ${p}_{ijk}$ is the probability that plaice *i* from unique deployment *j* within trip *k* is alive; ${X}_{1. ijk}.\dots .\kern0.5em {X}_{nijk}$represents the fixed effects of the models (here the vitality indicator tested and/or the environmental, technical and operational covariates) and ${\beta}_1$–${\beta}_n$their estimated coefficients.

One of the vitality indicators tested was the optimized version for the reflex impairment and injury index. In the ‘regular’ RI index (RI), all individual reflex and injuries attributes have an equal contribution to the overall index (weighting of 0.1 in our case based on a total of 10 reflexes and injuries) and RI is the resulting arithmetic mean. However, it is very likely that some reflex impairments or injuries have a higher predictive power than others. In the optimized version of the reflex impairment and injury index, each reflex and injury has its own weight which can be optimized to obtain the lowest AIC (on fish level) or the lowest prediction error in a linear model that compares fitted versus observed values on trip level (see later). In the optimized RI index (RI_optimized_), all individual reflex and injuries have a different contribution to the overall index and RI_optimized_ is the weighted mean where the weights are an indicator of the importance of each reflex or injury. Default starting values for the weighing of the reflex and injury attributes were set to 0.1, which corresponds to the regular RI (10 reflexes and injuries; values between 0 and 1 take 0.1 levels).

**Figure 1:**
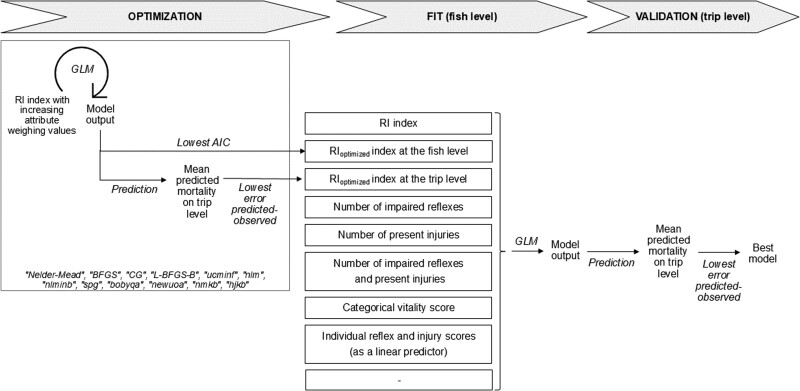
Scheme of the analytical approach to compare the ability of seven different vitality indicators and a null model (“-”) with and without biological, environmental, technical and operational covariates, to predict survival of fish at the trip level. To improve survival predictions by minimizing the error term, two approaches were evaluated by using logistic regression models: at individual fish level (lowest AIC) or aggregated as averages at trip level (model with smallest error terms between predicted and observed).

The performance of different optimization functions in optimizing the weights of individual reflex and injuries attributes was evaluated. This led to the optimized reflex impairment and injury (RI_optimized_) index with optimized weights for each of the six reflexes and four injuries. All GLM models were fitted on fish level, but choice of the best predictive models was examined at either individual fish level or at trip level (Fig. 1). For fish-level comparison, models that converged were ranked based on lowest Akaike Information Criterion (AIC) (Akaike, 1981). For trip-level comparisons, the absolute difference between the predicted mean survival on trip level (based on the individual predictions of all fish of that trip) and observed mean survival per trip was calculated.

Thus, the optimization procedure aimed at finding the weighing of the reflex and injury attributes that minimize the AIC for fish-level comparisons and the absolute difference between predicted and observed for trip-level comparisons. Optimization procedures were applied using the open-source R package OptimX (which include the following optimization methods that were used: ‘Nelder–Mead’, ‘BFGS’, ‘CG’, ‘L-BFGS-B’, ‘ucminf’, ‘nlm’, ‘nlminb’, ‘spg’, ‘bobyqa’, ‘newuoa’, ‘nmkb’, ‘hjkb’; Nash, 2022). If the models converged and if the results (i.e. the weightings) of each of these different optimization procedures were comparable amongst each other, the results could be trusted. Coefficients of individual reflex or injury types below the average (i.e. ≤ 0.10), between > 0.10 and ≤ 0.20 and > 0.20 were considered to indicate little, medium or high relevance in contributing to survival. Large coefficients > 0.30 with high optimization times were considered outliers. The optimization procedure resulted in two sets of optimized values for the weighing of the reflex and injury attributes, one that minimizes the AIC for fish-level comparisons (RI_optimized_ at fish level) and one that minimizes the absolute difference between predicted and observed for trip-level comparisons (RI_optimized_ at trip level).

The performance of the RI_optimized_ index at fish and trip levels was compared to five other vitality indicators, as well as reflex and injury types as individual indicators (partitioned) and a constant estimate, resulting in nine competing models with the following explanatory variables (Fig. 1):

Reflex impairment and injury index with equal weights (RI index), calculated as the mean score of all impaired reflexes and present injuries, which resulted in values ranging between 0 (representing an unimpaired and uninjured fish) and 1 (an impaired and injured fish),

RI_optimized_ index at fish level,

RI_optimized_ index at trip level,

Number of impaired reflexes,

Number of present injuries,

Number of impaired reflexes and present injuries, calculated as the sum of all the impaired reflexes and present injuries measured,

Categorical vitality score,

Reflex and injury types as individual indicators (partitioned),

Constant (null model).

All GLM models were fitted on fish level first, similarly to the optimization procedure. The choice of best model (validation) was made at the trip level, i.e. lowest absolute difference between the predicted mean survival on trip level (based on the individual predictions of all fish of that trip) and observed mean survival per trip (Fig. 1). To discuss which vitality indicator performed best for discard survival and for model validation, for each best combination of vitality indicator and/or explanatory variable(s), we compared the Brier score (lower is better) of the candidate models to the Brier score of the null model, also called the Index of Prediction Accuracy (IPA, higher is better), using the open-source R package riskRegression (Gerds *et al*., 2023). We did not use cross-validation as poor model fits (see results) would not lead to building predictive model for new data.

**Figure 2:**
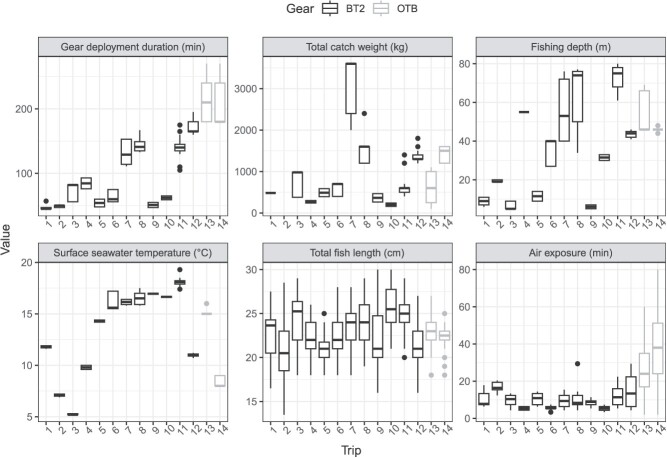
Gear deployment duration (min), total catch weights (kg), fishing depth (m), surface seawater temperature (°C), total fish length (cm) and air exposure (min) collected from European plaice (*Pleuronectes platessa*) during the 14 trips of Belgian commercial beam (BT2) and Danish otter trawlers (OTB).

**Figure 3:**
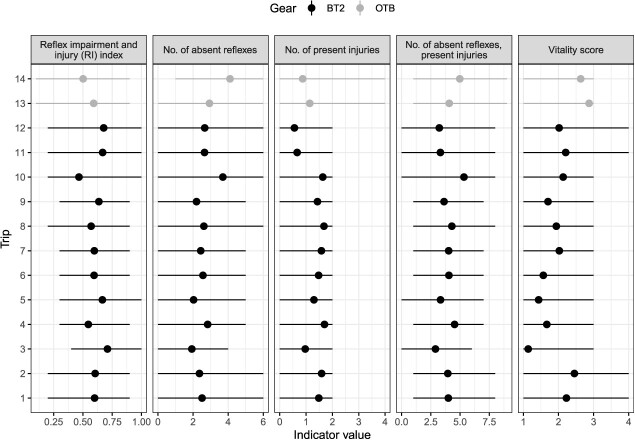
Values of reflex impairment and injury (RI) index (ranging between 0 and 1) of European plaice (*Pleuronectes platessa*), number of absent reflexes (between 0 and 6), number of present injuries (between 0 and 4), number of impaired reflexes and present injuries (between 0 and 10) and vitality score (considered here as a continuous variable from A as 1 to D as 4) given as min-max (line range) with mean (point) per trip for each gear type (Belgian beam trawl, BT2; or Danish otter trawl, OTB).

**Figure 4:**
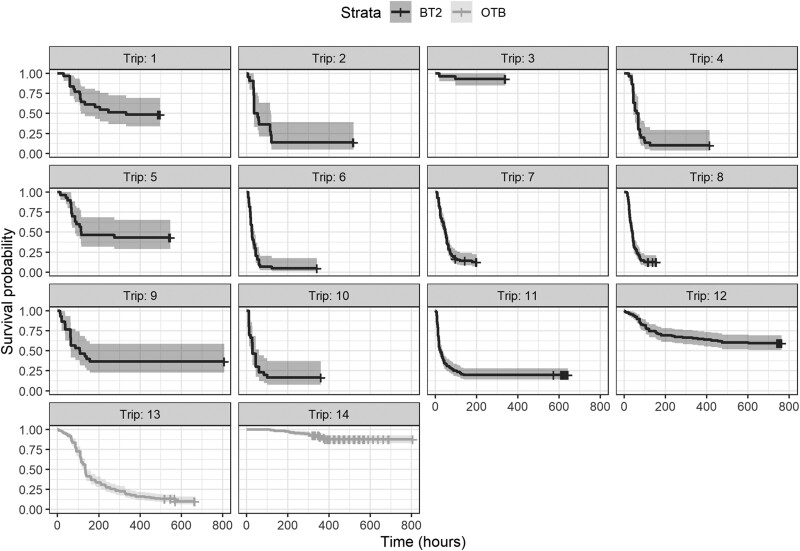
Non-parametric Kaplan–Meier survival probability estimates over days of monitoring until mortality reached asymptote of discarded European plaice (*Pleuronectes platessa*) collected during 12 trips with Belgian beam trawls (black line) and 2 trips with Danish otter trawls (grey line). Shaded areas around each line indicate 95% confidence intervals.

## Results

### Fishing operations

Undersized, trawl-caught plaice were collected from a total of 14 commercial fishing trips and 71 gear deployments. Belgian beam trawlers were monitored during 12 trips (57 deployments) between November 2014 and September 2015 as well as July and December 2020 in the eastern North Sea, eastern and western English Channel and north-western Atlantic Ocean. The Danish otter trawler conducted 2 trips (14 deployments) in the Western Baltic Sea between October 2020 and January 2021. Belgian beam trawlers targeted common sole between 5 and 80 m depth for trawl duration between 45 and 195 min, with surface seawater temperatures ranging between 5°C and 19°C (Fig. 2). The Danish otter trawler targeted plaice between 44 and 66 m depth for trawl duration between 180 and 270 min, with surface seawater temperatures ranging between 8 and 16°C (Fig. 2). Total catch weights were estimated at 1118 kg ± 874 kg and 674 kg ± 350 kg for Belgian beam and Danish otter trawlers, respectively (Fig. 2). Air exposures varied between 10 ± 5 min and 17 ± 18 min for beam- and otter-trawled fish, respectively (Fig. 2). Variation in air exposure is not directly proportional to catch weight as it also depends on catching and sorting procedures, including number of crew members.

In total, 675 and 392 undersized plaice (23 ± 2 cm TL, mean ± SD) were assessed for vitality and delayed survival from the monitored trips in Belgium and Denmark, respectively. For the vitality indicators, the average (i.e. aggregated across fish caught per trip) reflex impairment and injury between trips were similar, but reflex impairment was slightly greater in otter-trawl trips (Fig. 3). Survival at asymptote was reached between 6 and 34 days of captive observation, and some protracted mortality was observed (Fig. 4).

### Optimization of the reflex impairment and injury index

Model fits were applied to the data set cleaned from missing values with a total of 966 observations (missing values for total catch, TL and air exposure). At fish level, 97 out of 120 models converged with mean (min-max) optimization time of 0.21 s (0.00–1.54). At trip level, 113 out of 120 models converged with optimization time of 0.75 s (0.00–5.36) (Supporting information 1). All other models gave similar coefficients for a given optimization approach across the different optimization methods used and were thus considered accurate (Supporting information 1). For each covariate at fish or trip level, we present the best optimized model, i.e. lowest AIC for models at the fish and lowest error term for models at the trip levels, respectively (Table 2).

Overall, none of the individual reflex or injury indicators were independent of biological, environmental, technical and operational covariates when predicting plaice discard survival, both at fish and trip levels (Table 2). The best model for RI_optimized_ at fish (AIC = 857) and trip levels (error term: 0.095) included an interaction between gear and sea temperature (Table 2).

Bruising to the body was the most important contributor to the survival probability of discarded plaice, both at the fish and trip level, with weighing coefficients between 0.24 and 0.62 (Table 2) based on both the value of the weighing coefficients and how often these were below, in between or above the threshold levels (i.e. ≤ 0.10, between > 0.10 and ≤ 0.20 and > 0.20) across the different sets of covariates. The other important contributors were bruising to the head and tail grab at the fish level, and evasion, point to the head and bruising to the head at the trip level (Table 2). For the best model with the interaction of gear and sea surface temperature, at fish level, righting as well as bruising to the head and body were the most important contributors to the survival probability of discarded plaice with weighing coefficients for each attribute between 0.18 and 0.24 (Table 2). For the same model at the trip level, evasion, stabilize, bruising to the head and body contributed, with weighing coefficients between 0.12 and 0.33 (Table 2).

### Survival predictions

Vitality indicators need to be considered in combination with the biological, environmental, technical and operational variables, as all models showed a lower AIC score than the null model which ignores explanatory variables [Supporting information 2]). The best models (based on AIC) for each vitality indicator all included the interaction between gear and sea temperature. Model fits were applied to the data set cleaned from missing values for the two covariates of interest, i.e. gear and sea temperature, with a total of 1067 observations (no missing values).

**Figure 5:**
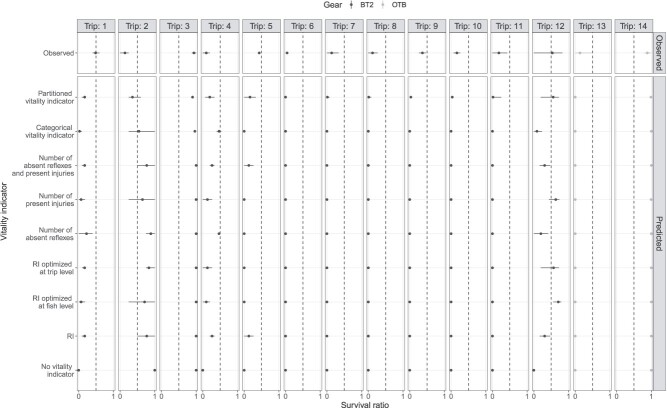
Comparison between observed and predicted survival ratio of European plaice (*Pleuronectes platessa*) given as min-max (line range) with mean (point) across deployments by trip and gear type (Belgian beam trawl, BT2; or Danish otter trawl, OTB). The 0.5 horizontal dash line is meant to discuss differences in predictions between high (>0.5) and low (<0.5) survival.

All of the models that include the interaction between gear and sea temperature for each vitality indicator showed a lower Brier score than the null model which ignores any vitality indicator variables (Table 3). The partitioned vitality indicator ranked best (Table 3). The RI_optimized_ index did not improve predictions markedly, whether it was optimized at the fish or the trip level (Table 3). Models with best metrics scores did not necessarily rank with best accuracy, e.g. highest accuracy for categorical vitality indicator (Table 3). All the vitality indicators were almost equally valuable proxies of plaice discard survival. Indeed, when we compared observed and predicted survival ratio for each gear (across trips) in the context of management purposes, i.e. assessing whether the survival ratio is high (>0.50) or low (≤0.50), all vitality indicators could correctly predict high or low survival (Table 3). This was also the case when no vitality indicator was used (Table 3). There were however discrepancies across trips for which not all vitality indicators could correctly predict high or low survival for trips 2 and 12 (Fig. 5).

**Table 2:** Coefficients of each reflex and injury attribute in the optimizing procedure of the reflex and injury index (RI_optimized_). The coefficients result from the best optimization method for the fish and trip levels selected using the model selection values of AIC or error term, respectively, and optimization time (in seconds). The weights are given without (NA) and with each covariate or combination of covariates (the biological, environmental, technical and operational explanatory variables). Coefficients with medium (>0.10 and ≤ 0.20) and high (>0.20) relevance in contributing to survival are in italics and bold, respectively, whereas those with little (≤0.10) contribution are presented with no typographic emphasis. Models are given ranked by model selection value (AIC at fish level and error term at trip level)

Covariates	Level	Method	Body flex	Righting	Head complex	Evasion	Stabilize	Tail grab	Bruising head	Bruising body	Point head	Point body	Model selection	Value	Optimization time
+Gear*SeaTemp	Fish	ucminf	*0.11*	*0.18*	0.04	0.02	0.10	0.08	0.22	0.24	0.00	0.00	AIC	857	0.04
+SeaTemp*AirExp		BFGS	0.10	*0.16*	*0.13*	0.03	0.05	*0.15*	*0.13*	0.26	0.00	0.00		874	0.00
+SeaTemp		ucminf	0.09	0.09	*0.12*	0.00	0.00	0.23	0.10	0.37	0.00	0.00		911	0.05
+MainWaterDepth		BFGS	0.09	0.00	0.00	0.00	0.00	0.05	0.28	0.58	0.00	0.00		1144	0.01
+AirExp		ucminf	0.00	0.02	0.00	0.00	0.00	*0.13*	*0.14*	0.37	0.22	*0.11*		1173	0.00
+Gear		nlminb	0.00	0.03	0.00	0.00	0.00	*0.17*	*0.18*	0.40	0.21	0.01		1210	0.03
+TotalCatch		BFGS	0.08	0.00	0.00	0.00	0.00	0.04	0.31	0.53	0.03	0.00		1212	0.02
+Length		bobyqa	0.05	0.00	0.00	0.00	0.00	0.07	0.29	0.53	0.06	0.00		1215	0.01
NA		spg	0.06	0.00	0.00	0.00	0.00	0.07	0.29	0.54	0.04	0.00		1217	0.04
+HaulDuration		ucminf	0.05	0.00	0.00	0.00	0.00	0.08	0.27	0.52	0.07	0.00		1219	0.03
+Gear*SeaTemp	Trip	Nelder–Mead	0.08	0.07	0.00	*0.16*	*0.12*	0.00	0.24	0.33	0.00	0.00	Error term	0.095	0.25
+SeaTemp*AirExp		nmkb	0.00	0.09	0.06	*0.20*	0.03	*0.15*	0.08	0.29	*0.11*	0.00		0.105	0.03
+SeaTemp		ucminf	0.00	0.00	0.00	0.28	0.04	0.00	0.03	0.31	0.25	0.09		0.117	0.10
+MainWaterDepth		spg	0.00	0.02	0.00	0.00	0.00	0.00	0.36	0.62	0.00	0.00		0.171	0.00
+AirExp		ucminf	0.00	0.09	0.00	0.10	0.00	0.03	0.09	0.29	0.29	0.10		0.201	0.17
+Length		Nelder–Mead	0.00	0.02	0.00	*0.19*	0.00	0.00	*0.20*	0.43	*0.17*	0.00		0.209	0.27
+Gear		nlm	0.00	0.10	0.00	*0.12*	0.00	0.04	*0.14*	0.37	0.24	0.00		0.217	0.06
NA		spg	0.00	0.08	0.00	*0.15*	0.00	0.00	0.21	0.44	*0.12*	0.00		0.218	0.08
+TotalCatch		spg	0.00	0.07	0.00	*0.14*	0.00	0.00	0.22	0.45	*0 .12*	0.00		0.220	0.13
+HaulDuration		nlm	0.04	0.05	0.00	0.09	0.00	0.00	0.30	0.52	0.00	0.00		0.221	0.05

**Table 3:** Best model fit (including an interaction between gear and sea temperature for each vitality indicator with score metrics presented as AIC (lower is better), Brier score (lower is better) and Index of Prediction Accuracy (IPA, higher is better) in bold for best model selection value. The confusion matrix displays the number of correctly predicted survivors (class 0, True Positives TP), the number of correctly predicted non-survivors (class 1, True Negatives TN), the number of survivors (class 0) incorrectly predicted as non-survivors (class 1, False Negatives FN) and the number of non-survivors (class 1) incorrectly predicted as survivors (class 0, False Positives FP). Accuracy (Acc.) is presented as (TP + TN)/(TP + TN + FP + FN). The survival ratio SR is given as mean (min-max) across trips by gear type (Belgian beam trawl, BT2; or Danish otter trawl, OTB)

Vitality indicator	AIC	Brier	IPA	TP	FP	FN	TN	Acc.	SR BT2	SR OTB
*Observed*	*-*	*-*	*-*	*-*	*-*	*-*	*-*	*-*	*0.31 (0.05–0.93)*	*0.50 (0.12–0.89)*
RI	973	13.9	39.9	237	150	51	629	0.81	0.22 (0.00–1.00)	0.50 (0.00–1.00)
RI_optimized_ at fish level	984	14.2	38.7	256	131	64	616	0.82	0.22 (0.00–1.00)	0.50 (0.00–1.00)
RI_optimized_ at trip level	988	14.2	38.4	254	133	59	621	0.82	0.23 (0.00–1.00)	0.50 (0.00–1.00)
No. absent reflexes	999	14.5	37.1	223	164	59	621	0.79	0.23 (0.00–1.00)	0.50 (0.00–1.00)
No. present injuries	992	14.3	38.1	253	134	55	625	0.82	0.21 (0.00–1.00)	0.50 (0.00–1.00)
No. absent reflexes and present injuries	973	13.9	39.9	237	150	51	629	0.81	0.22 (0.00–1.00)	0.50 (0.00–1.00)
Partitioned	947	13.2	42.9	211	176	43	637	0.79	0.21 (0.00–0.90)	0.50 (0.00–0.99)
Categorical	980	14.3	38.0	256	131	53	627	0.83	0.17 (0.00–0.97)	0.50 (0.00–1.00)
Null model	1029	23.1	0.00	198	189	42	638	0.78	0.17 (0.00–1.00)	0.50 (0.00–1.00)

## Discussion

This study showed for the first time that it is possible to create an optimized reflex and injury index with differential weights of each reflex and injury attribute included in the index. The most consistent predictor of plaice discard survival was bruising to the body and head, in line with comparable discard survival studies of plaice (Depestele *et al*., 2014; Uhlmann *et al*., 2016; Savina *et al*., 2019). Such haemorrhaging, for example to a vital organ such as the brain, can seriously compromise the health of plaice regardless of environmental conditions. The disadvantages of scoring bleeding injury are its potential for observer bias and a time delay in becoming fully visible (Meeremans *et al*., 2017).

Surprisingly, optimisation of the reflex impairment and injury index did not improve predictions. In fact, neither vitality indicators nor biological, environmental, technical or operational variables alone were accurate predictors of plaice discard survival at either fish or trip level. Comparable studies using whole-organism vitality indices to predict post-release survival of fish have faced similar uncertainties in predictions (Kraak *et al*., 2019). The combination of vitality indicators with technical/operational and environmental variables such as gear or seawater temperature was more important and improved the predictability. This suggests that a key underlying assumption of independence of vitality indicators as proxies from biological, environmental or experimental conditions in correlation with survival might not be met for trawl-caught-and-discarded plaice. Alternatively, if vitality indicators do integrate the effects of the different stressors, the importance of the covariates may be in their ability to create external conditions during which an individual should be able to recover. This recovery ability may be linked to the condition of the fish, which is currently not measured in discard survival studies.

Nevertheless, in contrast to other studies (Uhlmann *et al*., 2016, 2021; Van Der Reijden *et al*., 2017), assessment of either the categorical vitality score or injury/reflex alone did not necessarily correlate with post-capture survival. The reflex impairment and injury index and the categorical vitality score were almost equally valuable indicators of plaice discard survival (at least at correct prediction of high or low survival). Based on the same finding, Morfin *et al*. (2019) suggested that the most cost-effective method of categorizing vitality is suitable when rolled out as part of an ongoing data collection observer programme where vitality information is recorded alongside environmental conditions to discover possible correlations between both observations (Braccini *et al*., 2012; Falco *et al.*, 2022).

Both in Morfin *et al*. (2019) and in our study, contrasts between vitality conditions of individual fish disappeared when index scores were aggregated to groups of fish (i.e. per trip) because most fish had at least some impaired reflexes and injuries. Survival predictions then centre around a mean rate rather than around observed values for specific reflexes or injuries. Here, the lack of contrast in the distribution of vitality index scores between trips contributed to a slightly higher accuracy of fish-level than trip-level survival predictions of plaice (IPA). Use of a vitality indicator performed better than without it, but there was very little difference in the overall prediction of survival rates. However, the noise that remains when vitality indices are included in prediction models without including environmental factors demonstrated that cause–effect relationships between stressors and reflex impairment or occurrence of injury remain poorly understood within the reflex impairment framework. The assumption that reflex impairment and injury were directly related to stressor types and intensities (Breen and Catchpole, 2021) does not hold, because some covariates had to be included to improve fit. Maybe reflex impairment and injury do reflect the combined impact of all stressors and describe well the condition of the fish but is badly correlated to survival because the ability of the fish to recover is dependent on the environmental conditions.

Replicated exposure trials across single stressor gradients are needed to unravel any synergistic, antagonistic or simple cumulative effects of interactions among stressors (Raby *et al*., 2012; Uhlmann *et al*., 2016). These results indicated that despite a relationship between vitality and survival, and contrary to its underlying cause-and-effect theory, not all stressor effects resulted in reflex impairment and injury. This could mean that either some fish were more stressed and injured than what was captured by external visual and tactile examination; or other relevant, explanatory factors were not measured but which were in fact relevant contributors to survival (e.g. catch composition to quantify the effect of abrasive and injury-inducing elements present inside the catch; Savina *et al*., 2019; Uhlmann *et al.*, 2023). Vitality was clearly not the only explanatory variable when fitted to survival probability. Seawater surface temperature and gear were relevant variables as well.

Previous research corroborated that elevated temperatures compromise a species’ and an individual’s resilience towards commercial fishing capture (e.g. Davis, 2002; Gale *et al*., 2011, 2013; Uhlmann *et al*., 2016; Kraak *et al*., 2019). Prolonged exposure throughout the catch-and-handling process as well as elevated temperatures trigger a higher metabolic rate and a depletion of energy reserves which in turn makes fish prone to respond deleteriously towards commercial fishing capture stress (Gale *et al*., 2011, 2013). Elevated temperatures increase the release of stress hormones (primary stress response; Wendelaar Bonga, 1997; Schreck, 2010), but this effect may be abated among some species and individuals by other factors. How the interplay of environmental factors and chronic stress during being held in captivity causes a differential activation of the brain-sympathetic-chromaffin cell axis and the brain-pituitary-interrenal axis among plaice and how it affects its metabolism, energy reserves, and eventually its chance to survive cannot be unravelled here. But it is clear that measurements of ambient, environmental temperature are critical for the interpretation of any vitality versus survival relationship.

Another factor which has the potential to confound results is the amount of air exposure (Methling *et al*., 2017) or time spent in water prior to visual (reflex) assessment (Van Der Reijden *et al*., 2017; Cook *et al*., 2019). It is well known that holding organisms in oxygen rich water can contribute to their recovery (Yochum *et al*., 2015; Uhlmann *et al*., 2016), which should be avoided when examining reflex responsiveness of animals after impact to minimize any confounding effect of research-related handling. Unfortunately, the exact dose–response relationships of stressors are often not known or measured for individual fish, which does not allow to account for it when making predictions of survival. In this study, the time plaice spent in water-filled containers prior to being visually scored differed to an unknown extent between Belgium and Denmark.

Our work illustrated that predictions of plaice discard survival based on vitality proxies are not robust and require caution when being used in management contexts (STECF, 2018, 2021) or as a catch welfare indicator (Breen *et al.*, 2020). “Borrowing” vitality observations from one study to another can generate misleading survival estimates, especially if context-specific conditions are not recorded and collected following comparable and harmonized protocols. We showed that predictions of discard survival probability of trawled-and-discarded plaice are less uncertain at individual fish than at trip level. We suggest that bruising to the body was the most important contributors to the survival probability of discarded plaice, and overall vitality indicators performed the best as predictors of discard survival when additional explanatory variables were considered. From a management point of view, a good indicator should be simple to apply and easily understood as well as acceptable in terms of costs. Therefore a partitioned or optimized RI with focus on bruising to the body or a categorical vitality score seem to be sensitive enough to indicate correctly whether survival is high or low, but more accurate predictions will need to look at variability at the trip level. The methodology that was developed here can be used to be applied in other studies where vitality indicators are used to predict discard survival, in either commercial or recreational fisheries, but also in any other study using semi-qualitative scoring such as catch quality.

## Supplementary Material

Web_Material_coae070

## Data Availability

The data underlying this article are available in the article and in its online supplementary material.

## Footnotes

1 impairment absent = 0; impairment present = 1
^2^ injury absent = 0; injury present = 1
