# Supplementary material for: Optimizing the prediction of discard survival of bottom-trawled plaice based on vitality indicators
Source: Conserv Physiol. 2024 Oct 9;12(1):coae070. doi: 10.1093/conphys/coae070 (PMC11464240; doi:10.1093/conphys/coae070)
Supplement: Web_Material_coae070 [file web_material_coae070.zip › Supporting_information.pdf]

Supporting information

Supporting information 1. Estimated weights of each reflex and injury attribute in the optimizing procedure of the reflex and injury index ($RI_{optimized}$). The weights (or coefficients) are given per optimization methods (for detailed information, see the help pages of the open-source R package OptimX; Nash et al., 2022). The estimates are given without (NA) and with the different covariates or their one-way interactions at the fish level and trip level with their model selection values of AIC and error term, respectively, whether.the model converged and the model's optimization time in seconds. Weightings threshold are to be interpreted based on an average value of 0.10 if all reflexes and injuries have an equal weighting (ten scores were collected per individual fish), i.e., coefficients with medium (>0.10 and ≤ 0.20) and high (>0.20) relevance in contributing to survival against those with little (≤0.10) contribution.

Covariates	Optimization. level	Method	Body.flex	Righting	Head.complex	Evasion	Stabilise	Tail.grab	Bruising.head	Bruising.body	Point.head	Point.body	Model.selection. value	Convergence	Optimization. time
NA	AIC	spg	0.06	0	0	0	0	0.07	0.29	0.54	0.04	0	1217.25	Yes	0.04
NA	AIC	bobyqa	0.06	0	0	0	0	0.07	0.29	0.54	0.04	0	1217.25	Yes	0.05
NA	AIC	nlm	0.06	0	0	0	0	0.07	0.29	0.54	0.04	0	1217.25	Yes	0.06
NA	AIC	ucminf	0.06	0	0	0	0	0.07	0.29	0.54	0.04	0	1217.25	Yes	0.06
NA	AIC	newuoa	0.06	0	0	0	0	0.07	0.29	0.54	0.04	0	1217.25	Yes	0.09
NA	AIC	Nelder-Mead	0.06	0	0	0	0	0.07	0.29	0.54	0.04	0	1217.25	Yes	0.19
NA	AIC	hjkb	0.06	0	0	0	0	0.07	0.29	0.54	0.04	0	1217.25	Yes	0.3
NA	AIC	BFGS	0.06	0	0	0	0	0.07	0.29	0.54	0.04	0	1217.25	Yes	0.33
NA	AIC	nmkb	0.06	0	0	0	0	0.07	0.29	0.54	0.04	0	1217.25	Yes	0.42
NA	AIC	nlminb	0.06	0	0	0	0	0.07	0.29	0.54	0.04	0	1217.25	No	0.03
NA	AIC	CG	0.06	0	0	0	0	0.07	0.29	0.54	0.04	0	1217.25	No	35.81
NA	AIC	L-BFGS-B	0.06	0	0	0	0	0.07	0.29	0.54	0.04	0	1217.25	No	0.33
NA	tripmeans	hjkb	0	0.08	0	0.15	0	0	0.21	0.44	0.12	0	0.22	Yes	1.88

NA	tripmeans	nlminb	0	0.08	0	0.15	0	0	0.21	0.44	0.12	0	0.22	Yes	0.36
NA	tripmeans	nlm	0	0.08	0	0.15	0	0	0.21	0.44	0.12	0	0.22	Yes	0.53
NA	tripmeans	newuoa	0	0.08	0	0.15	0	0	0.21	0.44	0.12	0	0.22	Yes	3.39
NA	tripmeans	CG	0	0.08	0	0.15	0	0	0.21	0.44	0.12	0	0.22	Yes	0.78
NA	tripmeans	BFGS	0	0.08	0	0.15	0	0	0.21	0.44	0.12	0	0.22	Yes	0.34
NA	tripmeans	Nelder-Mead	0	0.08	0	0.15	0	0	0.21	0.44	0.12	0	0.22	Yes	0.34
NA	tripmeans	nmkb	0	0.08	0	0.15	0	0	0.21	0.44	0.12	0	0.22	Yes	0.46
NA	tripmeans	spg	0	0.08	0	0.15	0	0	0.21	0.44	0.12	0	0.22	Yes	0.08
NA	tripmeans	bobyqa	0	0.08	0	0.15	0	0	0.21	0.44	0.12	0	0.22	Yes	0.11
NA	tripmeans	ucminf	0	0.08	0	0.15	0	0	0.21	0.44	0.12	0	0.22	Yes	0.16
NA	tripmeans	L-BFGS-B	0	0.08	0	0.15	0	0	0.21	0.44	0.12	0	0.22	No	0.58
+Gear	AIC	newuoa	0	0.03	0	0	0	0.16	0.18	0.4	0.21	0.01	1209.67	Yes	0.24
+Gear	AIC	bobyqa	0	0.03	0	0	0	0.16	0.18	0.4	0.21	0.01	1209.67	Yes	0.09
+Gear	AIC	nlminb	0	0.03	0	0	0	0.16	0.18	0.4	0.21	0.01	1209.67	Yes	0.03
+Gear	AIC	hjkb	0	0.03	0	0	0	0.16	0.18	0.4	0.21	0.01	1209.67	Yes	0.88
+Gear	AIC	BFGS	0	0.03	0	0	0	0.16	0.18	0.4	0.21	0.01	1209.67	Yes	0.03
+Gear	AIC	spg	0	0.03	0	0	0	0.16	0.18	0.4	0.21	0.01	1209.67	Yes	0.13
+Gear	AIC	ucminf	0	0.03	0	0	0	0.16	0.18	0.4	0.21	0.01	1209.67	Yes	0.16
+Gear	AIC	Nelder-Mead	0	0.03	0	0	0	0.16	0.18	0.4	0.21	0.01	1209.67	Yes	0.17
+Gear	AIC	nlm	0	0.03	0	0	0	0.16	0.18	0.4	0.21	0.01	1209.67	Yes	0.24
+Gear	AIC	nmkb	0	0.03	0	0	0	0.16	0.18	0.4	0.21	0.01	1209.67	Yes	0.5
+Gear	AIC	CG	0	0.03	0	0	0	0.16	0.18	0.4	0.21	0.01	1209.67	No	45.83
+Gear	AIC	L-BFGS-B	0	0.03	0	0	0	0.16	0.18	0.4	0.21	0.01	1209.67	No	0.58
+Gear	tripmeans	bobyqa	0	0.1	0	0.12	0	0.04	0.14	0.36	0.24	0	0.22	Yes	0.08
+Gear	tripmeans	hjkb	0	0.1	0	0.12	0	0.04	0.14	0.36	0.24	0	0.22	Yes	1.75
+Gear	tripmeans	nlminb	0	0.1	0	0.12	0	0.04	0.14	0.36	0.24	0	0.22	Yes	0.09
+Gear	tripmeans	nlm	0	0.1	0	0.12	0	0.04	0.14	0.36	0.24	0	0.22	Yes	0.06
+Gear	tripmeans	ucminf	0	0.1	0	0.12	0	0.04	0.14	0.36	0.24	0	0.22	Yes	0.11

+Gear	tripmeans	BFGS	0	0.1	0	0.12	0	0.04	0.14	0.36	0.24	0	0.22	Yes	0.14
+Gear	tripmeans	spg	0	0.1	0	0.12	0	0.04	0.14	0.36	0.24	0	0.22	Yes	0.17
+Gear	tripmeans	newuoa	0	0.1	0	0.12	0	0.04	0.14	0.36	0.24	0	0.22	Yes	0.2
+Gear	tripmeans	nmkb	0	0.1	0	0.12	0	0.04	0.14	0.36	0.24	0	0.22	Yes	0.47
+Gear	tripmeans	Nelder-Mead	0	0.1	0	0.12	0	0.04	0.14	0.36	0.24	0	0.22	Yes	0.56
+Gear	tripmeans	CG	0	0.1	0	0.12	0	0.04	0.14	0.36	0.24	0	0.22	No	83.03
+Gear	tripmeans	L-BFGS-B	0	0.1	0	0.12	0	0.04	0.14	0.36	0.24	0	0.22	No	0.66
+HaulDuration	AIC	nlminb	0.05	0	0	0	0	0.08	0.27	0.52	0.07	0	1218.77	Yes	0.08
+HaulDuration	AIC	ucminf	0.05	0	0	0	0	0.08	0.27	0.52	0.07	0	1218.77	Yes	0.03
+HaulDuration	AIC	bobyqa	0.05	0	0	0	0	0.08	0.27	0.52	0.07	0	1218.77	Yes	0.03
+HaulDuration	AIC	BFGS	0.05	0	0	0	0	0.08	0.27	0.52	0.07	0	1218.77	Yes	0.04
+HaulDuration	AIC	spg	0.05	0	0	0	0	0.08	0.27	0.52	0.07	0	1218.77	Yes	0.09
+HaulDuration	AIC	nlm	0.05	0	0	0	0	0.08	0.27	0.52	0.07	0	1218.77	Yes	0.1
+HaulDuration	AIC	Nelder-Mead	0.05	0	0	0	0	0.08	0.27	0.52	0.07	0	1218.77	Yes	0.17
+HaulDuration	AIC	newuoa	0.05	0	0	0	0	0.08	0.27	0.52	0.07	0	1218.77	Yes	0.18
+HaulDuration	AIC	nmkb	0.05	0	0	0	0	0.08	0.27	0.52	0.07	0	1218.77	Yes	0.64
+HaulDuration	AIC	hjkb	0.05	0	0	0	0	0.08	0.27	0.52	0.07	0	1218.77	Yes	0.78
+HaulDuration	AIC	CG	0.05	0	0	0	0	0.08	0.27	0.52	0.07	0	1218.77	No	36.99
+HaulDuration	AIC	L-BFGS-B	0.05	0	0	0	0	0.08	0.27	0.52	0.07	0	1218.77	No	0.26
+HaulDuration	tripmeans	hjkb	0.04	0.05	0	0.09	0	0	0.3	0.52	0	0	0.22	Yes	1.29
+HaulDuration	tripmeans	nlminb	0.04	0.05	0	0.09	0	0	0.3	0.52	0	0	0.22	Yes	0.14
+HaulDuration	tripmeans	newuoa	0.04	0.05	0	0.09	0	0	0.3	0.52	0	0	0.22	Yes	0.5

+HaulDuration	tripmeans	Nelder-Mead	0.04	0.05	0	0.09	0	0	0.3	0.52	0	0	0.22	Yes	0.47
+HaulDuration	tripmeans	bobyqa	0.04	0.05	0	0.09	0	0	0.3	0.52	0	0	0.22	Yes	0.17
+HaulDuration	tripmeans	BFGS	0.04	0.05	0	0.09	0	0	0.3	0.52	0	0	0.22	Yes	0.1
+HaulDuration	tripmeans	spg	0.04	0.05	0	0.09	0	0	0.3	0.52	0	0	0.22	Yes	0.21
+HaulDuration	tripmeans	nlm	0.04	0.05	0	0.09	0	0	0.3	0.52	0	0	0.22	Yes	0.05
+HaulDuration	tripmeans	ucminf	0.04	0.05	0	0.09	0	0	0.3	0.52	0	0	0.22	Yes	0.12
+HaulDuration	tripmeans	nmkb	0.04	0.05	0	0.09	0	0	0.3	0.52	0	0	0.22	Yes	0.27
+HaulDuration	tripmeans	CG	0.04	0.05	0	0.09	0	0	0.3	0.52	0	0	0.22	No	93.99
+HaulDuration	tripmeans	L-BFGS-B	0.04	0.05	0	0.09	0	0	0.3	0.52	0	0	0.22	No	1
+TotalCatch	AIC	BFGS	0.08	0	0	0	0	0.04	0.31	0.53	0.03	0	1212.4	Yes	0.02
+TotalCatch	AIC	spg	0.08	0	0	0	0	0.04	0.31	0.53	0.03	0	1212.4	Yes	0.03
+TotalCatch	AIC	nlm	0.08	0	0	0	0	0.04	0.31	0.53	0.03	0	1212.4	Yes	0.08
+TotalCatch	AIC	bobyqa	0.08	0	0	0	0	0.04	0.31	0.53	0.03	0	1212.4	Yes	0.11
+TotalCatch	AIC	ucminf	0.08	0	0	0	0	0.04	0.31	0.53	0.03	0	1212.4	Yes	0.12
+TotalCatch	AIC	newuoa	0.08	0	0	0	0	0.04	0.31	0.53	0.03	0	1212.4	Yes	0.12
+TotalCatch	AIC	Nelder-Mead	0.08	0	0	0	0	0.04	0.31	0.53	0.03	0	1212.4	Yes	0.2
+TotalCatch	AIC	nmkb	0.08	0	0	0	0	0.04	0.31	0.53	0.03	0	1212.4	Yes	0.29
+TotalCatch	AIC	hjkb	0.08	0	0	0	0	0.04	0.31	0.53	0.03	0	1212.4	Yes	1
+TotalCatch	AIC	nlminb	0.08	0	0	0	0	0.04	0.31	0.53	0.03	0	1212.4	No	0.12
+TotalCatch	AIC	CG	0.08	0	0	0	0	0.04	0.31	0.53	0.03	0	1212.4	No	38.37
+TotalCatch	AIC	L-BFGS-B	0.08	0	0	0	0	0.04	0.31	0.53	0.03	0	1212.4	No	0.47
+TotalCatch	tripmeans	bobyqa	0	0.08	0	0.14	0	0	0.22	0.44	0.12	0	0.22	Yes	0.25
+TotalCatch	tripmeans	newuoa	0	0.08	0	0.14	0	0	0.22	0.44	0.12	0	0.22	Yes	0.17
+TotalCatch	tripmeans	hjkb	0	0.08	0	0.14	0	0	0.22	0.44	0.12	0	0.22	Yes	2.57
+TotalCatch	tripmeans	nlminb	0	0.08	0	0.14	0	0	0.22	0.44	0.12	0	0.22	Yes	0.9

+TotalCatch	tripmeans	L-BFGS-B	0	0.08	0	0.14	0	0	0.22	0.44	0.12	0	0.22	Yes	0.62
+TotalCatch	tripmeans	ucminf	0	0.08	0	0.14	0	0	0.22	0.44	0.12	0	0.22	Yes	0.42
+TotalCatch	tripmeans	nlm	0	0.08	0	0.14	0	0	0.22	0.44	0.12	0	0.22	Yes	0.52
+TotalCatch	tripmeans	CG	0	0.08	0	0.14	0	0	0.22	0.44	0.12	0	0.22	Yes	5.36
+TotalCatch	tripmeans	spg	0	0.08	0	0.14	0	0	0.22	0.44	0.12	0	0.22	Yes	0.13
+TotalCatch	tripmeans	Nelder-Mead	0	0.08	0	0.14	0	0	0.22	0.44	0.12	0	0.22	Yes	0.86
+TotalCatch	tripmeans	BFGS	0	0.08	0	0.14	0	0	0.22	0.44	0.12	0	0.22	Yes	2.17
+TotalCatch	tripmeans	nmkb	0	0.08	0	0.14	0	0	0.22	0.44	0.12	0	0.22	Yes	0.31
+SeaTemp	AIC	bobyqa	0.09	0.09	0.12	0	0	0.23	0.1	0.37	0	0	911.4	Yes	0.13
+SeaTemp	AIC	spg	0.09	0.09	0.12	0	0	0.23	0.1	0.37	0	0	911.4	Yes	0.1
+SeaTemp	AIC	nlminb	0.09	0.09	0.12	0	0	0.23	0.1	0.37	0	0	911.4	Yes	0.22
+SeaTemp	AIC	hjkb	0.09	0.09	0.12	0	0	0.23	0.1	0.37	0	0	911.4	Yes	0.83
+SeaTemp	AIC	nlm	0.09	0.09	0.12	0	0	0.23	0.1	0.37	0	0	911.4	Yes	0.11
+SeaTemp	AIC	CG	0.09	0.09	0.12	0	0	0.23	0.1	0.37	0	0	911.4	Yes	1.54
+SeaTemp	AIC	BFGS	0.09	0.09	0.12	0	0	0.23	0.1	0.37	0	0	911.4	Yes	0.11
+SeaTemp	AIC	Nelder-Mead	0.09	0.09	0.12	0	0	0.23	0.1	0.37	0	0	911.4	Yes	0.41
+SeaTemp	AIC	newuoa	0.09	0.09	0.12	0	0	0.23	0.1	0.37	0	0	911.4	Yes	0.26
+SeaTemp	AIC	ucminf	0.09	0.09	0.12	0	0	0.23	0.1	0.37	0	0	911.4	Yes	0.05
+SeaTemp	AIC	nmkb	0.09	0.09	0.12	0	0	0.23	0.1	0.37	0	0	911.4	Yes	0.37
+SeaTemp	AIC	L-BFGS-B	0.09	0.09	0.12	0	0	0.23	0.1	0.37	0	0	911.4	No	0.81
+SeaTemp	tripmeans	bobyqa	0	0	0	0.33	0	0	0	0.38	0.29	0	0.12	Yes	0.64
+SeaTemp	tripmeans	newuoa	0	0	0	0.33	0	0	0	0.38	0.29	0	0.12	Yes	0.19
+SeaTemp	tripmeans	hjkb	0	0	0	0.33	0	0	0	0.38	0.29	0	0.12	Yes	1.03
+SeaTemp	tripmeans	CG	0	0	0	0.33	0	0	0	0.38	0.29	0	0.12	Yes	4.55
+SeaTemp	tripmeans	nlminb	0	0	0	0.33	0	0	0	0.38	0.29	0	0.12	Yes	0.87
+SeaTemp	tripmeans	L-BFGS-B	0	0	0	0.33	0	0	0	0.38	0.29	0	0.12	Yes	0.93
+SeaTemp	tripmeans	Nelder-Mead	0	0	0	0.33	0	0	0	0.38	0.29	0	0.12	Yes	0.8

+SeaTemp	tripmeans	BFGS	0	0	0	0.33	0	0	0	0.38	0.29	0	0.12	Yes	0.76
+SeaTemp	tripmeans	spg	0	0	0	0.33	0	0	0	0.38	0.29	0	0.12	Yes	0.37
+SeaTemp	tripmeans	nmkb	0	0	0	0.33	0	0	0	0.38	0.29	0	0.12	Yes	0.37
+SeaTemp	tripmeans	nlm	0	0	0	0.27	0	0	0	0.37	0.28	0.08	0.12	Yes	0.25
+SeaTemp	tripmeans	ucminf	0	0	0	0.28	0.04	0	0.03	0.31	0.25	0.09	0.12	Yes	0.1
+Length	AIC	bobyqa	0.05	0	0	0	0	0.07	0.29	0.53	0.06	0	1214.97	Yes	0.01
+Length	AIC	spg	0.05	0	0	0	0	0.07	0.29	0.53	0.06	0	1214.97	Yes	0.02
+Length	AIC	ucminf	0.05	0	0	0	0	0.07	0.29	0.53	0.06	0	1214.97	Yes	0.03
+Length	AIC	BFGS	0.05	0	0	0	0	0.07	0.29	0.53	0.06	0	1214.97	Yes	0.05
+Length	AIC	nlm	0.05	0	0	0	0	0.07	0.29	0.53	0.06	0	1214.97	Yes	0.06
+Length	AIC	Nelder-Mead	0.05	0	0	0	0	0.07	0.29	0.53	0.06	0	1214.97	Yes	0.1
+Length	AIC	newuoa	0.05	0	0	0	0	0.07	0.29	0.53	0.06	0	1214.97	Yes	0.14
+Length	AIC	nmkb	0.05	0	0	0	0	0.07	0.29	0.53	0.06	0	1214.97	Yes	0.66
+Length	AIC	hjkb	0.05	0	0	0	0	0.07	0.29	0.53	0.06	0	1214.97	Yes	0.72
+Length	AIC	nlminb	0.05	0	0	0	0	0.07	0.29	0.53	0.06	0	1214.97	No	0.03
+Length	AIC	CG	0.05	0	0	0	0	0.07	0.29	0.53	0.06	0	1214.97	No	34.69
+Length	AIC	L-BFGS-B	0.05	0	0	0	0	0.07	0.29	0.53	0.06	0	1214.97	No	0.49
+Length	tripmeans	bobyqa	0	0.02	0	0.19	0	0	0.2	0.43	0.17	0	0.21	Yes	0.28
+Length	tripmeans	nlminb	0	0.02	0	0.19	0	0	0.2	0.43	0.17	0	0.21	Yes	0.9
+Length	tripmeans	hjkb	0	0.02	0	0.19	0	0	0.2	0.43	0.17	0	0.21	Yes	2.91
+Length	tripmeans	newuoa	0	0.02	0	0.19	0	0	0.2	0.43	0.17	0	0.21	Yes	1.81
+Length	tripmeans	nlm	0	0.02	0	0.19	0	0	0.2	0.43	0.17	0	0.21	Yes	0.87
+Length	tripmeans	ucminf	0	0.02	0	0.19	0	0	0.2	0.43	0.17	0	0.21	Yes	0.69
+Length	tripmeans	L-BFGS-B	0	0.02	0	0.19	0	0	0.2	0.43	0.17	0	0.21	Yes	0.52
+Length	tripmeans	CG	0	0.02	0	0.19	0	0	0.2	0.43	0.17	0	0.21	Yes	2.65
+Length	tripmeans	spg	0	0.02	0	0.19	0	0	0.2	0.43	0.17	0	0.21	Yes	0.38
+Length	tripmeans	Nelder-Mead	0	0.02	0	0.19	0	0	0.2	0.43	0.17	0	0.21	Yes	0.27
+Length	tripmeans	BFGS	0	0.02	0	0.19	0	0	0.2	0.43	0.17	0	0.21	Yes	1.06

+Length	tripmeans	nmkb	0	0	0	0.19	0	0	0.2	0.44	0.17	0	0.21	Yes	0.39
+AirExp	AIC	nlminb	0	0.02	0	0	0	0.13	0.14	0.37	0.22	0.11	1172.62	Yes	0.08
+AirExp	AIC	bobyqa	0	0.02	0	0	0	0.13	0.14	0.37	0.22	0.11	1172.62	Yes	0.03
+AirExp	AIC	ucminf	0	0.02	0	0	0	0.13	0.14	0.37	0.22	0.11	1172.62	Yes	0
+AirExp	AIC	newuoa	0	0.02	0	0	0	0.13	0.14	0.37	0.22	0.11	1172.62	Yes	0.06
+AirExp	AIC	BFGS	0	0.02	0	0	0	0.13	0.14	0.37	0.22	0.11	1172.62	Yes	0.09
+AirExp	AIC	spg	0	0.02	0	0	0	0.13	0.14	0.37	0.22	0.11	1172.62	Yes	0.11
+AirExp	AIC	nlm	0	0.02	0	0	0	0.13	0.14	0.37	0.22	0.11	1172.62	Yes	0.13
+AirExp	AIC	Nelder-Mead	0	0.02	0	0	0	0.13	0.14	0.37	0.22	0.11	1172.62	Yes	0.19
+AirExp	AIC	hjkb	0	0.02	0	0	0	0.13	0.14	0.37	0.22	0.11	1172.62	Yes	0.41
+AirExp	AIC	nmkb	0	0.02	0	0	0	0.13	0.14	0.37	0.22	0.11	1172.62	Yes	0.43
+AirExp	AIC	CG	0	0.02	0	0	0	0.13	0.14	0.37	0.22	0.11	1172.62	No	46.24
+AirExp	AIC	L-BFGS-B	0	0.02	0	0	0	0.13	0.14	0.37	0.22	0.11	1172.62	No	0.6
+AirExp	tripmeans	bobyqa	0	0.09	0	0.1	0	0.03	0.09	0.29	0.29	0.11	0.2	Yes	1.11
+AirExp	tripmeans	hjkb	0	0.09	0	0.1	0	0.03	0.09	0.29	0.29	0.11	0.2	Yes	2.49
+AirExp	tripmeans	nlm	0	0.09	0	0.1	0	0.03	0.09	0.29	0.29	0.11	0.2	Yes	0.28
+AirExp	tripmeans	nlminb	0	0.09	0	0.1	0	0.03	0.09	0.29	0.29	0.11	0.2	Yes	0.53
+AirExp	tripmeans	CG	0	0.09	0	0.1	0	0.03	0.09	0.29	0.29	0.11	0.2	Yes	0.93
+AirExp	tripmeans	BFGS	0	0.09	0	0.1	0	0.03	0.09	0.29	0.29	0.11	0.2	Yes	0.25
+AirExp	tripmeans	Nelder-Mead	0	0.09	0	0.1	0	0.03	0.09	0.29	0.29	0.11	0.2	Yes	0.24
+AirExp	tripmeans	spg	0	0.09	0	0.1	0	0.03	0.09	0.29	0.29	0.11	0.2	Yes	0.3
+AirExp	tripmeans	newuoa	0	0.09	0	0.1	0	0.03	0.09	0.29	0.29	0.1	0.2	Yes	0.81
+AirExp	tripmeans	ucminf	0	0.09	0	0.1	0	0.03	0.09	0.29	0.29	0.1	0.2	Yes	0.17
+AirExp	tripmeans	nmkb	0	0.09	0	0.1	0	0.03	0.09	0.29	0.29	0.1	0.2	Yes	0.33
+AirExp	tripmeans	L-BFGS-B	0	0.09	0	0.1	0	0.03	0.09	0.29	0.29	0.1	0.2	No	0.79
+MainWaterDepth	AIC	nlminb	0.09	0	0	0	0	0.05	0.28	0.58	0	0	1143.81	Yes	0.03
+MainWaterDepth	AIC	BFGS	0.09	0	0	0	0	0.05	0.28	0.58	0	0	1143.81	Yes	0.01

+MainWaterDepth	AIC	nlm	0.09	0	0	0	0	0.05	0.28	0.58	0	0	1143.81	Yes	0.03
+MainWaterDepth	AIC	spg	0.09	0	0	0	0	0.05	0.28	0.58	0	0	1143.81	Yes	0.04
+MainWaterDepth	AIC	bobyqa	0.09	0	0	0	0	0.05	0.28	0.58	0	0	1143.81	Yes	0.1
+MainWaterDepth	AIC	ucminf	0.09	0	0	0	0	0.05	0.28	0.58	0	0	1143.81	Yes	0.13
+MainWaterDepth	AIC	nmkb	0.09	0	0	0	0	0.05	0.28	0.58	0	0	1143.81	Yes	0.22
+MainWaterDepth	AIC	newuoa	0.09	0	0	0	0	0.05	0.28	0.58	0	0	1143.81	Yes	0.23
+MainWaterDepth	AIC	Nelder-Mead	0.09	0	0	0	0	0.05	0.28	0.58	0	0	1143.81	Yes	0.28
+MainWaterDepth	AIC	hjkb	0.09	0	0	0	0	0.05	0.28	0.58	0	0	1143.81	Yes	0.28
+MainWaterDepth	AIC	CG	0.09	0	0	0	0	0.05	0.28	0.58	0	0	1143.81	No	43.17
+MainWaterDepth	AIC	L-BFGS-B	0.09	0	0	0	0	0.05	0.28	0.58	0	0	1143.81	No	1.06
+MainWaterDepth	tripmeans	newuoa	0	0.02	0	0	0	0	0.36	0.62	0	0	0.17	Yes	0.54
+MainWaterDepth	tripmeans	hjkb	0	0.02	0	0	0	0	0.36	0.62	0	0	0.17	Yes	1.55
+MainWaterDepth	tripmeans	nlm	0	0.02	0	0	0	0	0.36	0.62	0	0	0.17	Yes	1.53
+MainWaterDepth	tripmeans	nlminb	0	0.02	0	0	0	0	0.36	0.62	0	0	0.17	Yes	0.23
+MainWaterDepth	tripmeans	CG	0	0.02	0	0	0	0	0.36	0.62	0	0	0.17	Yes	0.9
+MainWaterDepth	tripmeans	BFGS	0	0.02	0	0	0	0	0.36	0.62	0	0	0.17	Yes	0.2
+MainWaterDepth	tripmeans	Nelder-Mead	0	0.02	0	0	0	0	0.36	0.62	0	0	0.17	Yes	0.28
+MainWaterDepth	tripmeans	bobyqa	0	0.02	0	0	0	0	0.36	0.62	0	0	0.17	Yes	1.28
+MainWaterDepth	tripmeans	nmkb	0	0.02	0	0	0	0	0.36	0.62	0	0	0.17	Yes	0.49
+MainWaterDepth	tripmeans	spg	0	0.02	0	0	0	0	0.36	0.62	0	0	0.17	Yes	0
+MainWaterDepth	tripmeans	ucminf	0	0.02	0	0	0	0	0.36	0.62	0	0	0.17	Yes	0.28

+MainWaterDepth	tripmeans	L-BFGS-B	0	0.02	0	0	0	0	0.36	0.62	0	0	0.17	No	1.75
+Gear*SeaTemp	AIC	ucminf	0.11	0.18	0.04	0.02	0.1	0.08	0.22	0.24	0	0	856.63	Yes	0.04
+Gear*SeaTemp	AIC	nlm	0.11	0.18	0.04	0.02	0.1	0.08	0.22	0.24	0	0	856.63	Yes	0.06
+Gear*SeaTemp	AIC	spg	0.11	0.18	0.04	0.02	0.1	0.08	0.22	0.24	0	0	856.63	Yes	0.06
+Gear*SeaTemp	AIC	BFGS	0.11	0.18	0.04	0.02	0.1	0.08	0.22	0.24	0	0	856.63	Yes	0.06
+Gear*SeaTemp	AIC	bobyqa	0.11	0.18	0.04	0.02	0.1	0.08	0.22	0.24	0	0	856.63	Yes	0.08
+Gear*SeaTemp	AIC	newuoa	0.11	0.18	0.04	0.02	0.1	0.08	0.22	0.24	0	0	856.63	Yes	0.11
+Gear*SeaTemp	AIC	Nelder-Mead	0.11	0.18	0.04	0.02	0.1	0.08	0.22	0.24	0	0	856.63	Yes	0.2
+Gear*SeaTemp	AIC	hjkb	0.11	0.18	0.04	0.02	0.1	0.08	0.22	0.24	0	0	856.63	Yes	0.33
+Gear*SeaTemp	AIC	nmkb	0.11	0.18	0.04	0.02	0.1	0.08	0.22	0.24	0	0	856.63	Yes	0.34
+Gear*SeaTemp	AIC	nlminb	0.11	0.18	0.04	0.02	0.1	0.08	0.22	0.24	0	0	856.63	No	0.09
+Gear*SeaTemp	AIC	CG	0.11	0.18	0.04	0.02	0.1	0.08	0.22	0.24	0	0	856.63	No	38.29
+Gear*SeaTemp	AIC	L-BFGS-B	0.11	0.18	0.04	0.02	0.1	0.08	0.22	0.24	0	0	856.63	No	0.44
+Gear*SeaTemp	tripmeans	bobyqa	0.08	0.07	0	0.16	0.12	0	0.24	0.33	0	0	0.1	Yes	0.53
+Gear*SeaTemp	tripmeans	newuoa	0.08	0.07	0	0.16	0.12	0	0.24	0.33	0	0	0.1	Yes	0.28
+Gear*SeaTemp	tripmeans	nlminb	0.08	0.07	0	0.16	0.12	0	0.24	0.33	0	0	0.1	Yes	0.36
+Gear*SeaTemp	tripmeans	hjkb	0.08	0.07	0	0.16	0.12	0	0.24	0.33	0	0	0.1	Yes	2.21
+Gear*SeaTemp	tripmeans	ucminf	0.08	0.07	0	0.16	0.12	0	0.24	0.33	0	0	0.1	Yes	0.5
+Gear*SeaTemp	tripmeans	nlm	0.08	0.07	0	0.16	0.12	0	0.24	0.33	0	0	0.1	Yes	0.44
+Gear*SeaTemp	tripmeans	CG	0.08	0.07	0	0.16	0.12	0	0.24	0.33	0	0	0.1	Yes	1.69
+Gear*SeaTemp	tripmeans	spg	0.08	0.07	0	0.16	0.12	0	0.24	0.33	0	0	0.1	Yes	0.35

+Gear*SeaTemp	tripmeans	L-BFGS-B	0.08	0.07	0	0.16	0.12	0	0.24	0.33	0	0	0.1	Yes	0.26
+Gear*SeaTemp	tripmeans	Nelder-Mead	0.08	0.07	0	0.16	0.12	0	0.24	0.33	0	0	0.1	Yes	0.25
+Gear*SeaTemp	tripmeans	BFGS	0.08	0.07	0	0.16	0.12	0	0.24	0.33	0	0	0.1	Yes	0.47
+Gear*SeaTemp	tripmeans	nmkb	0.08	0.07	0	0.16	0.12	0	0.24	0.33	0	0	0.1	Yes	0.33
+SeaTemp*AirExp	AIC	nlminb	0.1	0.16	0.13	0.03	0.05	0.15	0.13	0.26	0	0	873.67	Yes	0.03
+SeaTemp*AirExp	AIC	BFGS	0.1	0.16	0.13	0.03	0.05	0.15	0.13	0.26	0	0	873.67	Yes	0
+SeaTemp*AirExp	AIC	nlm	0.1	0.16	0.13	0.03	0.05	0.15	0.13	0.26	0	0	873.67	Yes	0
+SeaTemp*AirExp	AIC	Nelder-Mead	0.1	0.16	0.13	0.03	0.05	0.15	0.13	0.26	0	0	873.67	Yes	0.02
+SeaTemp*AirExp	AIC	ucminf	0.1	0.16	0.13	0.03	0.05	0.15	0.13	0.26	0	0	873.67	Yes	0.05
+SeaTemp*AirExp	AIC	spg	0.1	0.16	0.13	0.03	0.05	0.15	0.13	0.26	0	0	873.67	Yes	0.09
+SeaTemp*AirExp	AIC	newuoa	0.1	0.16	0.13	0.03	0.05	0.15	0.13	0.26	0	0	873.67	Yes	0.1
+SeaTemp*AirExp	AIC	bobyqa	0.1	0.16	0.13	0.03	0.05	0.15	0.13	0.26	0	0	873.67	Yes	0.15
+SeaTemp*AirExp	AIC	hjkb	0.1	0.16	0.13	0.03	0.05	0.15	0.13	0.26	0	0	873.67	Yes	0.64
+SeaTemp*AirExp	AIC	nmkb	0.1	0.16	0.13	0.03	0.05	0.15	0.13	0.26	0	0	873.67	Yes	0.75
+SeaTemp*AirExp	AIC	CG	0.1	0.16	0.13	0.03	0.05	0.15	0.13	0.26	0	0	873.67	No	50.96
+SeaTemp*AirExp	AIC	L-BFGS-B	0.1	0.16	0.13	0.03	0.05	0.15	0.13	0.26	0	0	873.67	No	0.62
+SeaTemp*AirExp	tripmeans	bobyqa	0	0.09	0.06	0.2	0.03	0.14	0.07	0.29	0.11	0	0.11	Yes	0.11
+SeaTemp*AirExp	tripmeans	newuoa	0	0.09	0.06	0.2	0.03	0.14	0.07	0.29	0.11	0	0.11	Yes	0.24
+SeaTemp*AirExp	tripmeans	hjkb	0	0.09	0.06	0.2	0.03	0.14	0.07	0.29	0.11	0	0.11	Yes	3.12
+SeaTemp*AirExp	tripmeans	nlminb	0	0.09	0.06	0.2	0.03	0.14	0.07	0.29	0.11	0	0.11	Yes	0.86
+SeaTemp*AirExp	tripmeans	ucminf	0	0.09	0.06	0.2	0.03	0.14	0.07	0.29	0.11	0	0.11	Yes	0.33

+SeaTemp*AirExp	tripmeans	nlm	0	0.09	0.06	0.2	0.03	0.14	0.07	0.29	0.11	0	0.11	Yes	0.52
+SeaTemp*AirExp	tripmeans	L-BFGS-B	0	0.09	0.06	0.2	0.03	0.14	0.07	0.29	0.11	0	0.11	Yes	0.4
+SeaTemp*AirExp	tripmeans	spg	0	0.09	0.06	0.2	0.03	0.14	0.07	0.29	0.11	0	0.11	Yes	0.07
+SeaTemp*AirExp	tripmeans	CG	0	0.09	0.06	0.2	0.03	0.14	0.07	0.29	0.11	0	0.11	Yes	1.67
+SeaTemp*AirExp	tripmeans	BFGS	0	0.09	0.06	0.2	0.03	0.14	0.07	0.29	0.11	0	0.11	Yes	1.14
+SeaTemp*AirExp	tripmeans	Nelder-Mead	0	0.09	0.06	0.2	0.03	0.14	0.07	0.29	0.11	0	0.11	Yes	0.8
+SeaTemp*AirExp	tripmeans	nmkb	0	0.09	0.06	0.2	0.03	0.14	0.08	0.29	0.11	0	0.11	Yes	0.03

Supporting information 2. For each vitality indicator, AIC for models fitted with biological, environmental, technical, and operational explanatory variables (left to right on the top row). For each vitality indicator, the lowest AIC is in bold.

Vitality indicator	+AirExp	+Gear	+Gear*SeaTemp	+HaulDuration	+Length	+MainWaterDepth	+SeaTemp	+SeaTemp*AirExp	+TotalCatch	None
RI	1213	1254	**879**	1269	1281	1238	933	891	1285	1286
$RI_{optimized}$ at fish level	1173	1210	**857**	1219	1215	1144	911	874	1212	1217
$RI_{optimized}$ at trip level	1181	1217	**866**	1223	1228	1146	943	888	1223	1228
Number of absent reflexes	1227	1268	**897**	1279	1287	1240	947	904	1291	1292
Number of present injuries	1180	1219	**896**	1231	1232	1177	939	909	1234	1236
Number of absent reflexes and present injuries	1213	1254	**879**	1269	1281	1238	932	891	1285	1286
Individual scores (partitioned)	1182	1213	**863**	1219	1216	1135	928	887	1215	1217
Categorical vitality score	1166	1195	**893**	1238	1279	1244	938	901	1284	1285
Null model	1225	1266	**922**	1277	1286	1241	958	927	1290	1292
